# A New Method for Detecting Dehydration of the Human Body Using Non-Contact Millimeter Wave Radiometry

**DOI:** 10.3390/s24144461

**Published:** 2024-07-10

**Authors:** Amani Yousef Owda

**Affiliations:** Department of Natural, Engineering and Technology Sciences, Arab American University, Ramallah P600, Palestine; amani.owda@aaup.edu

**Keywords:** dehydration, diabetes, vomiting, diarrhea, passive sensing, millimeter wave

## Abstract

Dehydration is a common problem in the aging population. Medical professionals can detect dehydration using either blood or urine tests. This requires experimental tests in the lab as well as urine and blood samples to be obtained from the patients. This paper proposed 100 GHz millimeter wave radiometry for early detection of dehydration. Reflectance measurements were performed on healthy and dehydrated patients of both genders (120 males and 80 females) in the aging population. Based on the cause of dehydration, the patient groups were divided into three categories: (1) patients dehydrated due to less thirst sensation, (2) patients dehydrated due to illnesses (vomiting and diarrhea), and (3) patients dehydrated due to diabetes. Reflectance measurements were performed on eight locations: (1) the palm, (2) the back of the hand, (3) the fingers, (4) the inner wrist, (5) the outer wrist, (6) the volar side of the arm, (7) the dorsal surface of the arm, and (8) the elbow. Skin dehydrated due to vomiting and diarrhea was found to have lower reflectance at all the measurement locations compared with healthy and other types of dehydrated skin. The elbow region showed the highest difference in reflectance between healthy and dehydrated skin. This indicates that radiometric sensitivity is sufficient to detect dehydration in a few seconds. This will reduce the patient’s waiting time and the healthcare professional’s intervention time as well as allow early treatment of dehydration, thus avoiding admission to hospitals.

## 1. Introduction

Dehydration is the most common fluid problem in the elderly. It causes around 1.35 million deaths every year globally [1]. The reasons for elderly people being prone to dehydration are not fully understood. Age-related changes in total body water, thirst perception, and renal concentrating ability probably predispose them to dehydration. A healthy adult human body consists of an average of 60% water [2]. This percentage is subject to change with age and variation in the body mass index [3,4,5,6]. The shortage of water in the human body causes dehydration, which can range from mild to severe [7,8]. Maintaining a healthy percentage of water content is crucial for human survival, as it allows human organs to function well. The elderly and children are the most affected by dehydration [8]. The main causes of dehydration are (1) vomiting, (2) diarrhea, (3) less sensation of feeling thirsty due to age, (4) diabetes, and (5) frequent sweating, either due to physical exercise or hot weather [9,10,11,12,13].

The main signs of dehydration include tiredness, rapid heartbeat, smelly urine, dry mouth, and dry skin [9,14]. Dry skin is one of the most important indicators of dehydration [15]. Skin is the largest organ of the human body and consists of about 16% of the body mass and covers the internal organs of the human body [16,17]. Skin status is a reliable indicator of the body’s health, for example, its dehydration [18]. For a dehydrated body, it was reported in [19,20] that there is a direct relationship between skin characteristics such as water content reduction and skin stiffness and roughness.

Dehydration can cause many illnesses, as water plays an important role in maintaining the functions of other organs such as the lungs, brain, and kidneys [8]. Medical professionals can assess dehydration using either a blood test or urine test [21,22]. This requires experimental tests to be conducted in the lab as well as urine and blood samples to be obtained from the patients. In addition, severe dehydration requires hospital admission, as the patient needs to be treated using intravenous fluids [23,24]. This consumes time and money. A technique that could provide non-invasive early detection of dehydration without the need for blood samples or urine tests in the lab will be beneficial for healthcare professionals, patients, and the healthcare industry. In this research, radiometry will be used for the first time as a non-contact sensor to detect dehydration in elderly patients.

There are many techniques used to assess skin hydration in the literature [25]. Optical Raman Spectroscopy [26] shows an increase in the thickness of the epidermal layer of the skin due to adding water to the skin. Optical near-infrared spectroscopy (NIRS) [27] provides information about the level of hydration based on the spectrum absorption bands that are related to the water content of the skin. In addition, the study in [28] proposed a multi-wavelength optical sensor for measuring the dermal water content of porcine skin samples. In [29], authors used electrical capacitance to assess skin moisture before and after the application of a gel. In [30], the authors used electrical bioimpedance to assess the hydration level of the skin using nanomesh electrodes and the correlation between the impedance and the hydration level of the skin and found a negative correlation of −0.85. In [31], researchers used an electric method called SkinUp to measure oil and moisture levels in the skin. The SkinUp method showed higher sensitivity after the application of creams on the skin.

Millimeter wave (MMW) is a frequency band ranging from 30 to 300 GHz and has wavelengths ranging from 10.0 mm to 1.0 mm [32,33]. MMW radiation is very sensitive to variations in water content in the skin, and therefore, it has been recommended for use in non-invasive diagnosis of diseased skin via measuring either the reflectance or the relative complex permittivity of the skin using open-ended coaxial probes [34,35,36,37]. The MMW probe indicates a well-defined difference in the permittivity between healthy skin and burned skin [34,38]. Researchers in [35,36] used the MMW probe to distinguish between wet skin (skin after the addition of gel or water) and normal skin. In addition, [39] indicated a substantial difference in dielectric permittivity between healthy skin and skin mutated due to basal cell carcinoma. The main limitations of using open-ended probes are the technical difficulty of conducting the measurements, as it is very sensitive to the alignments, as well as maintaining constant pressure and being in direct contact with the skin [40]. Based on these limitations, researchers in [41,42] suggested radiometry as a non-contact sensor for use in non-invasive diagnosis of diseased skin, where the disease affects the water content of the skin.

In our previous research [17], a half-space electromagnetic model demonstrated the feasibility of using radiometry to distinguish between healthy skin and unhealthy skin, i.e., skin after the application of gel, skin with malignant lesions, and synthetic skin with different water contents. The model converted the relative complex permittivity values of the skin into emissivity values and indicated differences between healthy and unhealthy skin based on results obtained from the electromagnetic simulation. Ref. [33] demonstrated reflectance measurements of healthy skin of both genders. This study indicated differences in the reflectance of the skin between male and female participants. Ref. [40] presented a multilayer electromagnetic model for estimating the reflectance of human skin over the frequency band 30–100 GHz using the relative complex permittivity measurements in the literature. Ref. [41] demonstrated a feasibility study using a radiometer to distinguish between healthy skin and burn-damaged skin of a chicken phantom. The study showed differences in mean emissivity values between healthy and burn-damaged skin. Ref. [42] used a passive millimeter wave imaging system at 250 GHz to assess the feasibility of passive sensing technology to monitor wound healing underdressing materials of porcine skin samples. None of these studies involved reflectance measurements performed on dehydrated patients. The reflectance measurements presented in this paper demonstrated for the first time the feasibility of using a radiometer as a non-contact sensor to distinguish between healthy skin and dehydrated skin.

It is well known that dehydration affects the water content of the human body and the skin [9]. In response to this change, the skin presents signatures that can be measured using non-contact passive millimeter wave sensors. Therefore, this research aims to propose MMW radiometry for the first time to be used as a non-contact sensor for early detection of human body dehydration. This technique will allow early detection of dehydration in a few seconds, and more importantly, without contact with the human body and without taking any urine or blood samples. During the COVID-19 pandemic, it was challenging to care for elderly or child patients suffering from dehydration in hospitals or healthcare centers. This challenge motivates the researchers to think about new techniques for the early detection of dehydration. Therefore, this paper proposes MMW radiometry as a new technique for quick and fast detection of dehydration. This will reduce the waiting time for the patient and the intervention time of healthcare professionals. This allows the early treatment of dehydration and avoids admission to hospitals.

## 2. Materials and Methods

**Figure 1:**
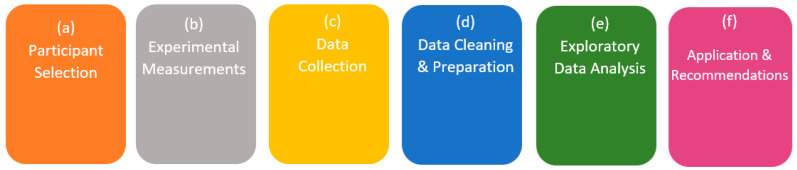
The methodology used in this research for conducting the experimental measurements on the participants. The methodology involved six main stages, namely participant selection (**a**); performing the experimental measurements (**b**); data collection and storing (**c**); data cleaning and data preparation (**d**); exploratory data analysis; (**e**) and application and recommendations (**f**).

The methodology used in this research involved experimental measurements performed on participants. The methodology consisted of six main stages, as illustrated in Figure 1. It starts with participant selection, as described in Section 2.1, after approval of the study under the ethics reference number of SE1617114C; this is followed by performing the experimental measurements on participants using radiometry, as explained in Section 2.3. Then, quantitative data collection and storage are carried out, as discussed in Section 2.3; this is followed by data cleaning and data preparation, as shown in Section 2.4. Exploratory data analysis is illustrated in Section 2.5, and finally, application of the method and further recommendations are described in Section 2.6.

### 2.1. Participant Selection and Description

The study was approved by the ethics committee under application reference number SE1617114C. Each participant was interviewed before performing the experimental measurements and agreed to sign the informed consent form. The participant information sheet was given to all participants and they were given time to read and ask questions. All patients who participated in this research are classified into dehydrated patients based on a urine test called the urine-specific gravity test or a blood test performed in medical labs. In addition, each patient had a medical report obtained from specialist doctors specifying the reason for dehydration. Reflectance measurements were conducted directly after the medical test and before the patient took any treatment or medication.

In this research, there were 120 male and 80 female participants divided into groups based on their health status. Table 1 shows the number of participants in each group of both genders, the mean (µ), and the standard deviation (SD) of the age in each group.

The selection of participants was based on participants who agreed to participate in this research. The researcher involved participants from the same age category for both genders. In addition, the measurements were performed on specific locations for all participants to minimize variation in reflectance due to age and varying location on the skin.

### 2.2. Experimental Setup and Selection of Measurement Locations

**Figure 2:**
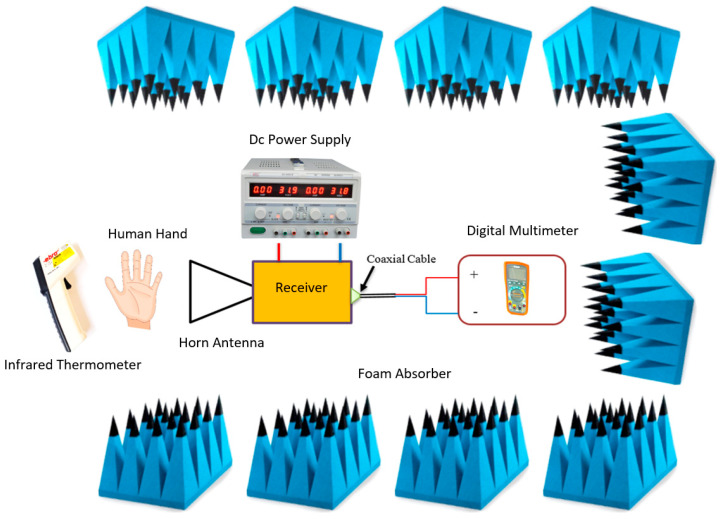
Experimental setup used for measuring the reflectance of the skin.

The experimental setup of measuring the reflectance of healthy and dehydrated skin is described in Figure 2. A direct detection MMW radiometer consisting of a horn antenna and receiver (type: direct detection; model MSi100; manufacturer: Digital Barriers, Glasgow, UK) was utilized for conducting the reflectance measurements on healthy and dehydrated skin. The direct detection receiver consists of a two-stage low-noise amplifier, zero bias diode detector, and buffer amplifier. The radiometer has a bandwidth of 10 GHz and a center frequency of 100 GHz. The radiometer was connected through wires to a DC power supply (model: DC 3005-II, Hazari Tech Connect, Maharashtra, India). The radiometer was calibrated using hot and cold calibration sources. The cold calibration source is a piece of MMW carbon-loaded foam absorber (model: Eccosorb AN-73; Laird, Shanghai, China) dipped in liquid nitrogen with a temperature of 77 K, whereas the hot calibration source is a piece of MMW carbon-loaded foam absorber with a temperature of 295 K.

The target area of the skin, i.e., hand and arm, was allocated over a distance of 4.0 cm from the W-band horn antenna (model number: AS4341; Atlan TecRF, Essex, UK). The antenna had a rectangular aperture of 25 mm × 20 mm, a bandwidth of 10 GHz, and a center frequency of 100 GHz. The selection of 100 GHz frequency was made for many reasons. (1) This frequency interacts mostly with the top 0.4 mm layer of the skin [32,33] and this means it is suited to the measurements of the epidermis and the dermis layers of the human skin rather than other internal organs. (2) This frequency is very sensitive to water molecules. (3) Millimeter wave devices (radiometers, horn antenna, and detectors) are widely available at this specific frequency, as they provide a balance between atmospheric absorption and spatial resolution [43].

A non-contact infrared thermometer (model number: TFI 260; Ebro, Beijing, China) with a resolution of 0.1 °C and an accuracy of 0.01 °C was used to measure the temperatures of the skin and the calibration sources. A digital multimeter (model number: EX350, Extech Instruments, Nashua, NH, USA) with a precision of 0.01 mV was used to measure the voltage level of the skin and the calibration sources. These measurements can be converted into reflectance using Equation (1) [33].

(1) $$R=\frac{T_{S}\left(V_{H}-V_{C}\right)+T_{H}\left(V_{C}-V_{S}\right)+T_{C}\left(V_{S}-V_{H}\right)}{\left(T_{s}-T_{H}\right)\left(V_{H}-V_{C}\right)}\;\;\;\;\;\;\;\;\;$$

where $T_{H}$, $T_{C}$, and $T_{S}$ are the temperatures in Kelvin of the hot calibration source, the cold calibration source, and the human skin, respectively. $V_{H},$ $V_{C}$, and $V_{S}$ are the voltage levels in millivolts of the thermal MMW emission from the hot calibration source, the cold calibration source, and the human skin, respectively.

The experimental setup was surrounded by MMW pyramidal carbon-loaded foam absorber (model number: SA-pyramidal foam absorber, EMC-PIONEER, Jiangsu, China) except for an opening for the target area of the skin to be measured. This prevented radiation from external sources from getting into the system and affecting the experimental measurements’ accuracy.

**Figure 3:**
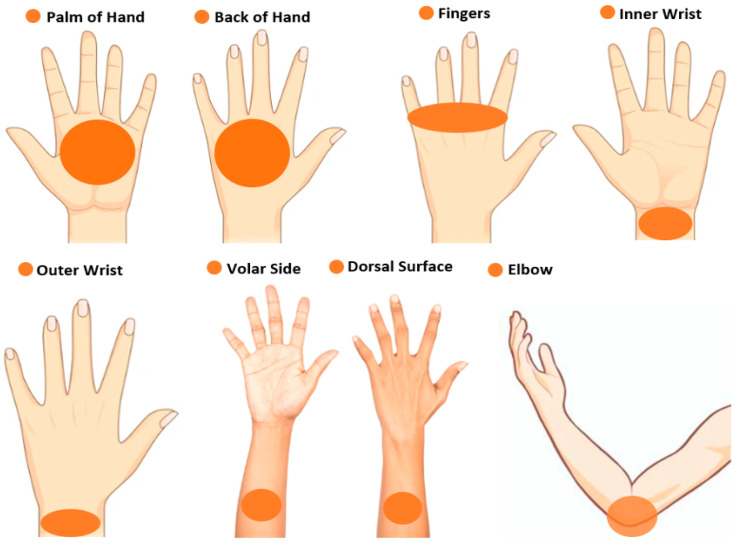
Measurement locations on the hand and arm were applied to all participants.

The experimental measurements were conducted in eight locations on the hand and arm, as illustrated in Figure 3. These locations are the palm, the back of the hand, the fingers, the inner wrist, the outer wrist, the volar side of the arm, the dorsal surface of the arm, and the elbow. These locations were chosen as there are no ethical issues associated with conducting measurements on these locations for both genders. In addition, it is easy to control and align the hand and the arm properly to fill the beam pattern of the horn antenna of the radiometer. This will reduce the uncertainty and generate accurate measurements of skin reflectance. The measurements were conducted in the near field zone. The target area of the skin was located over a distance of 4.0 cm from the horn antenna. This distance was chosen to minimize the chances of subjects accidentally touching and moving the experimental setup.

### 2.3. Data Collection

The voltage level and temperature of the skin and calibration sources were measured and each measurement was repeated five times. Then, the measurements were compared to make sure that they were consistent and the devices were stable. All identifiable information related to participants was anonymized. The data were saved in Excel files on a computer (OptiPlex 5040, Dell Technologies, Ramallah, Palestine). The computer was allocated in a research lab that could be accessed by members of the research group only. The Excel file contains eight subfiles for each measurement location. Each of those subfiles contains 10 columns (participant ID, age, gender, health status, temperature of the skin (location specified), temperature for hot calibration source, temperature for cold calibration source, voltage level of the skin (location specified), voltage level for hot calibration source, voltage level for cold calibration source) and 1000 rows, as each measurement was repeated five times.

### 2.4. Data Cleaning and Data Preparation

Data cleaning and data perpetration were carried out in parallel with the experimental work to maintain all participant findings. The voltage level and temperature measurements obtained from participants and calibration sources were converted into reflectance using Equation (1). All participants’ measurements were double-checked during the experimental work. This was to make sure that there were no missing values. Each measurement was repeated five times and compared to make sure that there were no wrong values. Considering these steps during the experimental work will allow us to use all participants’ measurements.

### 2.5. Exploratory Data Analysis

The exploratory data analysis (EDA) of reflectance measurements is based on data visualization and statistical analysis. A boxplot was used to represent all reflectance measurements of healthy and dehydrated skin. This visualization was chosen as it contains five summary points of reflectance measurements, and more importantly, it shows the distribution of the reflectance [44]. In addition, the reader can easily pick up outliers and make comparisons between healthy and dehydrated skin. Three reasons for dehydration were explored for male and female groups, and they are (1) dehydration due to less thirst sensation, (2) dehydration due to illnesses, and (3) dehydration due to diabetes. For the statistical analysis, the mean and the standard deviation were calculated for all measurement locations of both healthy and dehydrated skin groups. All these findings are presented and discussed in Section 3, i.e., results.

### 2.6. Application and Recommendation

The MMW radiometer used in this research indicated well-defined differences in reflectance between healthy and dehydrated skin. The dehydrated skin was classified based on medical reports obtained from a specialist doctor and the radiometric measurements were conducted before the patient took any treatments. The importance of the proposed technique, i.e., radiometer, is that it can measure the reflectance of the skin in a few seconds and without touching the human skin (non-contact). In addition, radiometry is classified as a passive sensor, as it collects thermal emissions without exposing the skin to any type of man-made radiation [40,45]. Based on the aforementioned facts, this research recommended the use of MMW radiometry as a new non-invasive technique for detecting skin dehydration.

## 3. Results

This section presents reflectance results obtained from male and female groups of healthy and dehydrated skin of all measurement locations described in the methodology in Section 2.2. A boxplot is used for making comparisons in reflectance between healthy and dehydrated skin regions, as illustrated in Section 3.1 and Section 3.2, respectively.

### 3.1. Male Sample Results

This section shows reflectance measurements on eight measurement locations of healthy and dehydrated skin of 120 male participants (30 healthy and 90 dehydrated due to less sensation of thirst, illnesses (vomiting and diarrhea), and diabetes, in which each of the dehydrated groups consisted of 30 participants). The results are presented in four subsections: Section 3.1.1—male participants dehydrated due to less thirst sensation, Section 3.1.2—male participants dehydrated due to illnesses (vomiting and diarrhea), and Section 3.1.3—male participants dehydrated due to diabetes.

#### 3.1.1. Male Participants Dehydrated Due to Less Thirst Sensation 

This section presents reflectance measurements of healthy male participants and male participants dehydrated due to less thirst sensation. The statement “less thirst sensation” means that the patient is dehydrated and the reason for dehydration is based on a lack of thirst sensation, and this is one of the negative impacts of aging, as reported by a specialist doctor.

**Figure 4:**
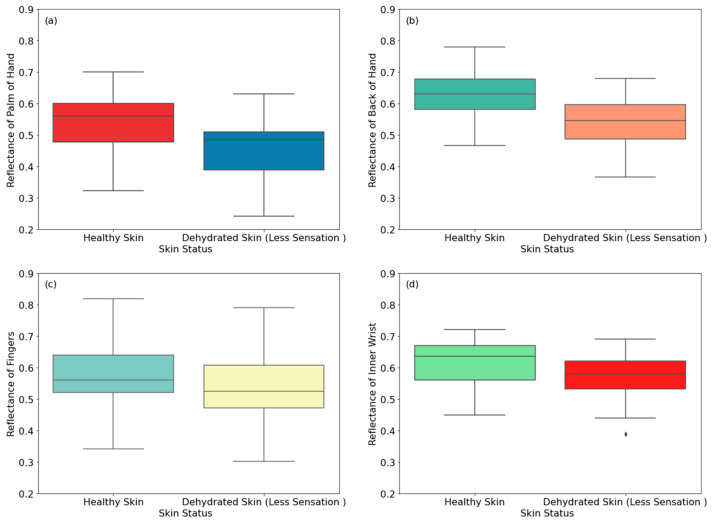

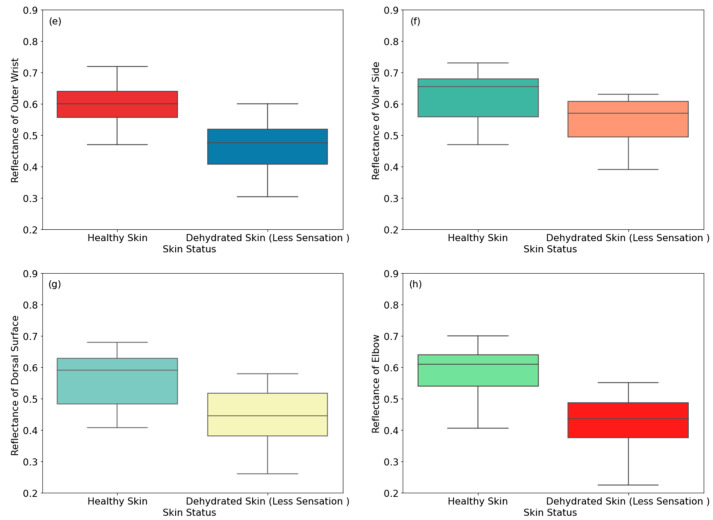
Reflectance measurements of healthy and dehydrated skin (due to less thirst sensation) for male participants on the palm (**a**), the back of the hand (**b**), the fingers (**c**), the inner wrist (**d**), the outer wrist (**e**), the volar side of the arm (**f**), the dorsal surface of the arm (**g**), and the elbow (**h**). The symbol (♦) indicates an exceptional data point (outlier).

The measurements in Figure 4 show reflectance from 30 healthy and 30 dehydrated male participants. The measurements were applied on eight regions of the skin, namely the palm, the back of the hand, the fingers, the inner wrist, the outer wrist, the volar side of the arm, the dorsal surface of the arm, and the elbow.

Figure 4 shows that the reflectance of dehydrated skin (due to less thirst sensation) is lower than that of healthy skin. The differences in the reflectance are 0.079, 0.085, 0.041, 0.042, 0.132, 0.079, 0.112, and 0.167 for the palm, the back of the hand, the fingers, inner wrist, outer wrist, volar side of the arm, dorsal surface of the arm, and the elbow, respectively. These differences are due to a lower water content of dehydrated skin compared with healthy skin [20,46]. Experimental measurements indicate that the highest difference in the reflectance between healthy and dehydrated skin is 16.7%, achieved on the elbow region of the arm, whereas the lowest difference in the reflectance is 4.1%, achieved on the finger region of the hand.

#### 3.1.2. Male Participants Dehydrated Due to Illnesses (Vomiting and Diarrhea)

**Figure 5:**
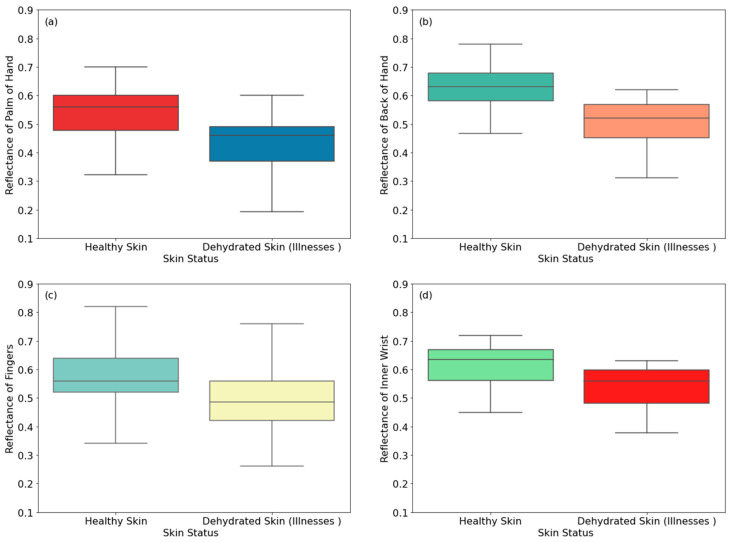

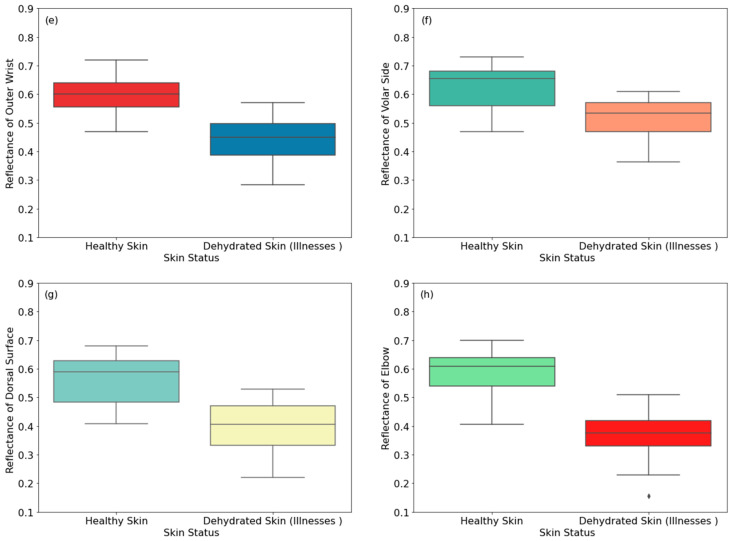
Reflectance measurements of healthy and dehydrated skin (due to vomiting and diarrhea) for male participants on the palm (**a**), the back of the hand (**b**), the fingers (**c**), the inner wrist (**d**), the outer wrist (**e**), the volar side of the arm (**f**), the dorsal surface of the arm (**g**), and the elbow (**h**). The symbol (♦) indicates an exceptional data point (outlier).

This section presents reflectance measurements of 30 healthy male participants and 30 dehydrated male participants suffering from vomiting and diarrhea, as illustrated in Figure 5.

Figure 5 shows that the reflectance of dehydrated skin (due to vomiting and diarrhea) is lower than that of healthy skin for all measurement locations. The differences in the reflectance are 0.110, 0.118, 0.082, 0.075, 0.160, 0.108, 0.156, and 0.220 for the palm, the back of the hand, the fingers, the inner wrist, the outer wrist, the volar side of the arm, the dorsal surface of the arm, and the elbow, respectively. These differences are due to lower water content of dehydrated skin compared with healthy skin [20,46]. Experimental measurements indicate that the highest difference in the reflectance between healthy and dehydrated skin is 22.0%, achieved on the elbow region of the arm, whereas the lowest difference in the reflectance is 7.5%, achieved on the inner wrist region of the hand. The measurements in Figure 5 indicate higher differences in the reflectance due to vomiting and diarrhea compared with Figure 4, which presents dehydration due to less sensation of thirst. This can be explained by the fact that less sensation of thirst can be controlled by providing elderly people with sufficient fluids such as water, juice, and soup [47,48]. On the other hand, participants suffering from vomiting and diarrhea are patients suffering from less thirst sensation due to their age. This makes the dehydration severe for this group of participants, and as a result, produces more differences in the reflectance between healthy and dehydrated skin regions.

#### 3.1.3. Male Participants Dehydrated Due to Diabetes

This section presents reflectance measurements of healthy and dehydrated male participants due to diabetes Type 2. The statement “patients with diabetes” means that the patient is dehydrated and the reason for dehydration is based on increased cumulative blood sugar (Hba1c), as reported by a specialist doctor.

**Figure 6:**
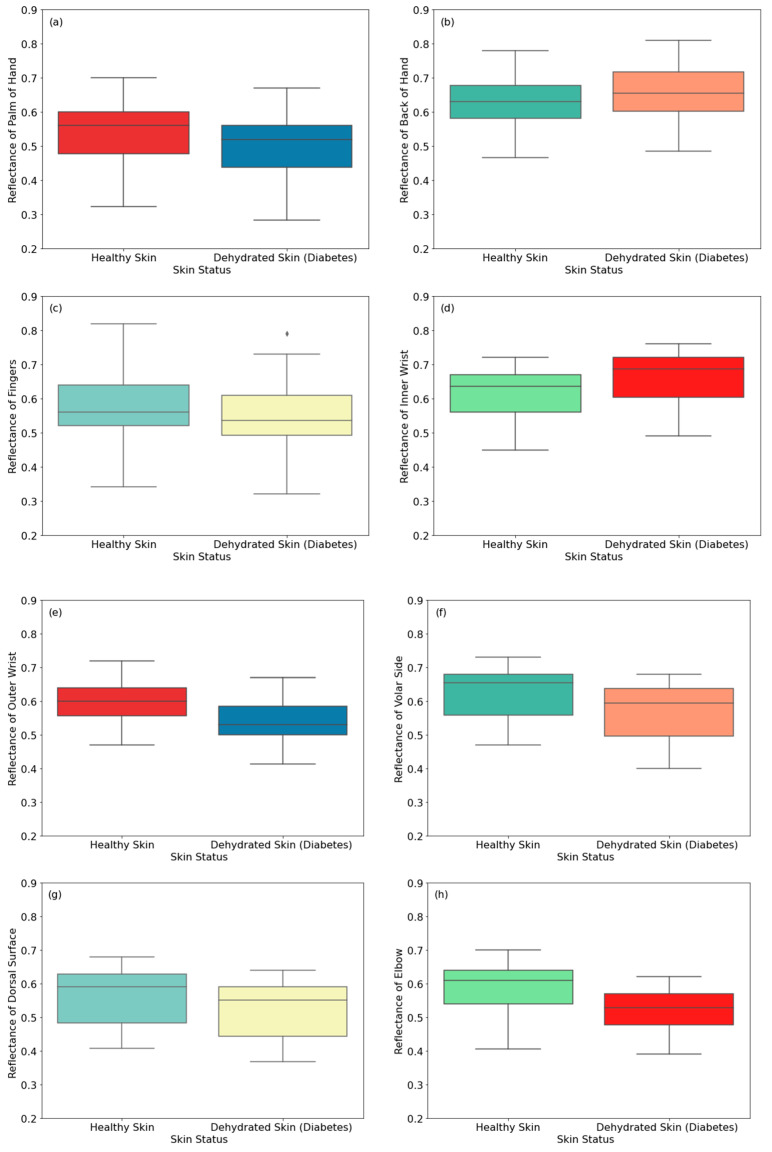
Reflectance measurements of healthy and dehydrated skin (due to diabetes) for male participants on the palm (**a**), the back of the hand (**b**), the fingers (**c**), the inner wrist (**d**), the outer wrist (**e**), the volar side of the arm (**f**), the dorsal surface of the arm (**g**), and the elbow (**h**). The symbol (♦) indicates an exceptional data point (outlier).

The measurements in Figure 6 show reflectance from 30 healthy and 30 dehydrated male participants with Type 2 diabetes. The measurements were applied to eight regions of the skin. Experimental measurements of the reflectance on the back of the hand and inner wrist regions of dehydrated skin are higher than those of healthy skin by 0.030 and 0.048, respectively. This is due to diabetes affecting skin thickness [49,50,51]. This affects human skin reflectance, as it varies with skin thickness [33,41]. Thinner skin regions present higher reflectance, as the blood vessels become closer to the skin surface [33]. On the other hand, the reflectance of dehydrated skin regions of the palm, fingers, outer wrist, volar side of the arm, dorsal surface of the arm, and the elbow is lower than that of healthy skin by 0.038, 0.025, 0.059, 0.052, 0.035, and 0.070, respectively.

### 3.2. Female Sample Results

This section shows reflectance measurements on eight measurement locations of healthy and dehydrated skin of 80 female participants (20 healthy and 60 dehydrated due to less sensation of thirst, illnesses (vomiting and diarrhea), and diabetes, in which each of the dehydrated group consisted of 20 participants). The results are presented in four sub-sections: Section 3.2.1—female participants dehydrated due to less thirst sensation, Section 3.2.2—female participants dehydrated due to illnesses (vomiting and diarrhea), and Section 3.2.3—female participants dehydrated due to diabetes.

#### 3.2.1. Female Participants Dehydrated Due to Less Thirst Sensation 

**Figure 7:**
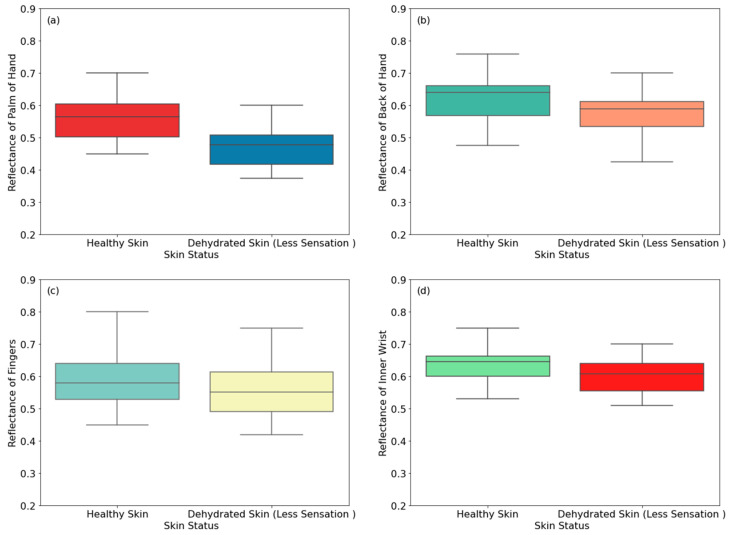

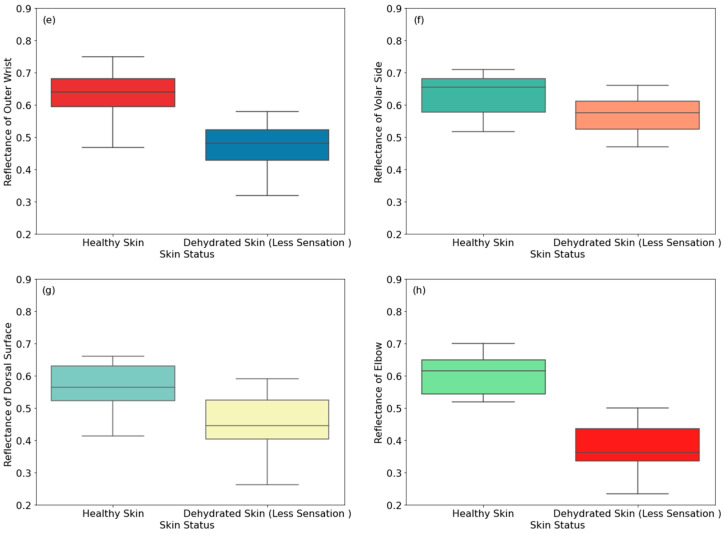
Reflectance measurements of healthy and dehydrated skin (due to less thirst sensation) for female participants on the palm (**a**), the back of the hand (**b**), the fingers (**c**), the inner wrist (**d**), the outer wrist (**e**), the volar side of the arm (**f**), the dorsal surface of the arm (**g**), and the elbow (**h**).

This section presents reflectance measurements of healthy female participants and female participants dehydrated due to less thirst sensation. The measurements in Figure 7 show reflectance from 20 healthy and 20 dehydrated female participants. The measurements were applied on eight regions of the skin, namely the palm, the back of the hand, the fingers, the inner wrist, the outer wrist, the volar side of the arm, the dorsal surface of the arm, and the elbow.

Figure 7 shows that the reflectance of dehydrated skin (due to less thirst sensation) is lower than that of healthy skin. The differences in the reflectance are 0.093, 0.053, 0.032, 0.034, 0.159, 0.063, 0.117, and 0.22 for the palm, the back of the hand, the fingers, the inner wrist, the outer wrist, the volar side of the arm, the dorsal surface of the arm, and the elbow, respectively. These differences are due to lower water content of dehydrated skin compared with healthy skin [20,46]. Experimental measurements indicate that the highest difference in the reflectance between healthy and dehydrated skin is 22%, achieved on the elbow region, whereas the lowest difference in the reflectance is 3.2%, achieved on the finger region. These results are consistent with the results presented in Figure 4 for male participants.

#### 3.2.2. Female Participants Dehydrated Due to Illnesses (Vomiting and Diarrhea)

**Figure 8:**
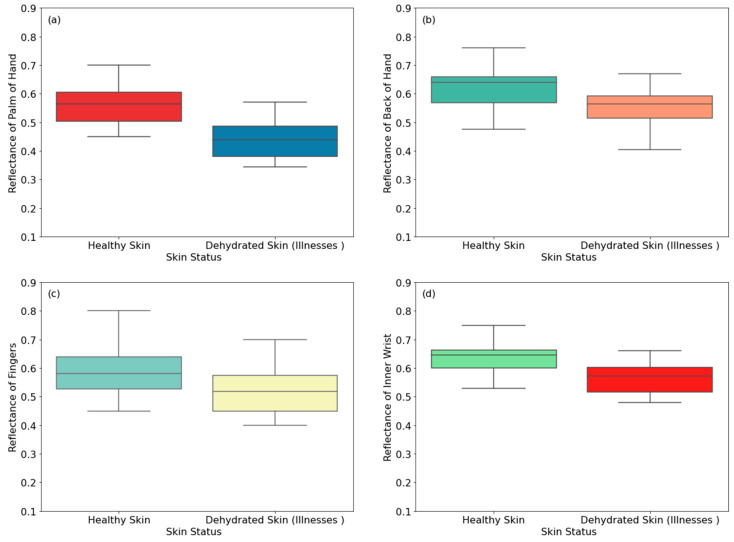

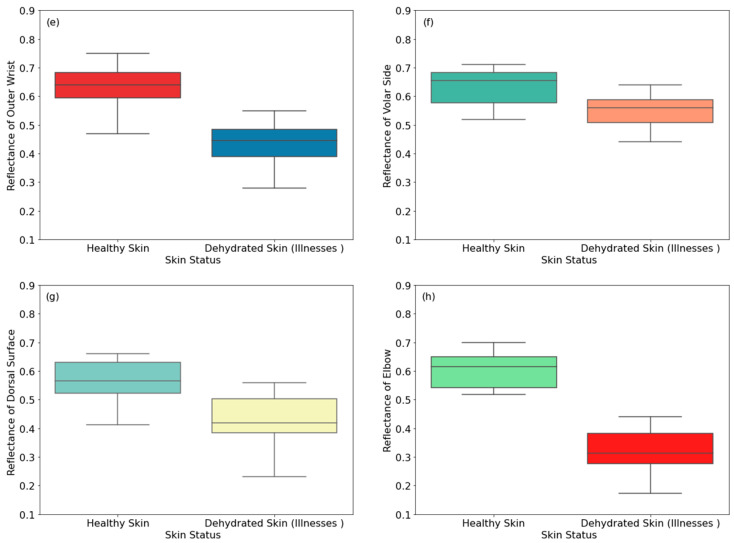
Reflectance measurements of healthy and dehydrated skin (due to illnesses) for female participants on the palm (**a**), the back of the hand (**b**), the fingers (**c**), the inner wrist (**d**), the outer wrist (**e**), the volar side of the arm (**f**), the dorsal surface of the arm (**g**), and the elbow (**h**).

This section presents reflectance measurements of 20 healthy and 20 dehydrated female participants suffering from vomiting and diarrhea, as illustrated in Figure 8.

Figure 8 shows that the reflectance of dehydrated skin (due to vomiting and diarrhea) is lower than that of healthy skin for all measurement locations. The differences in the reflectance are 0.122, 0.076, 0.060, 0.068, 0.195, 0.083, 0.143, and 0.274 for the palm of the hand, the back of the hand, the fingers, inner wrist, outer wrist, volar side of the arm, dorsal surface of the arm, and the elbow, respectively. These differences are due to a lower water content of dehydrated skin compared with healthy skin [20,46]. Experimental measurements indicate that the highest difference in the reflectance between healthy and dehydrated skin is 27.4%, achieved on the elbow region of the arm, whereas the lowest difference in the reflectance is 6.0%, achieved on the finger region of the hand.

#### 3.2.3. Female Participants Dehydrated Due to Diabetes

**Figure 9:**
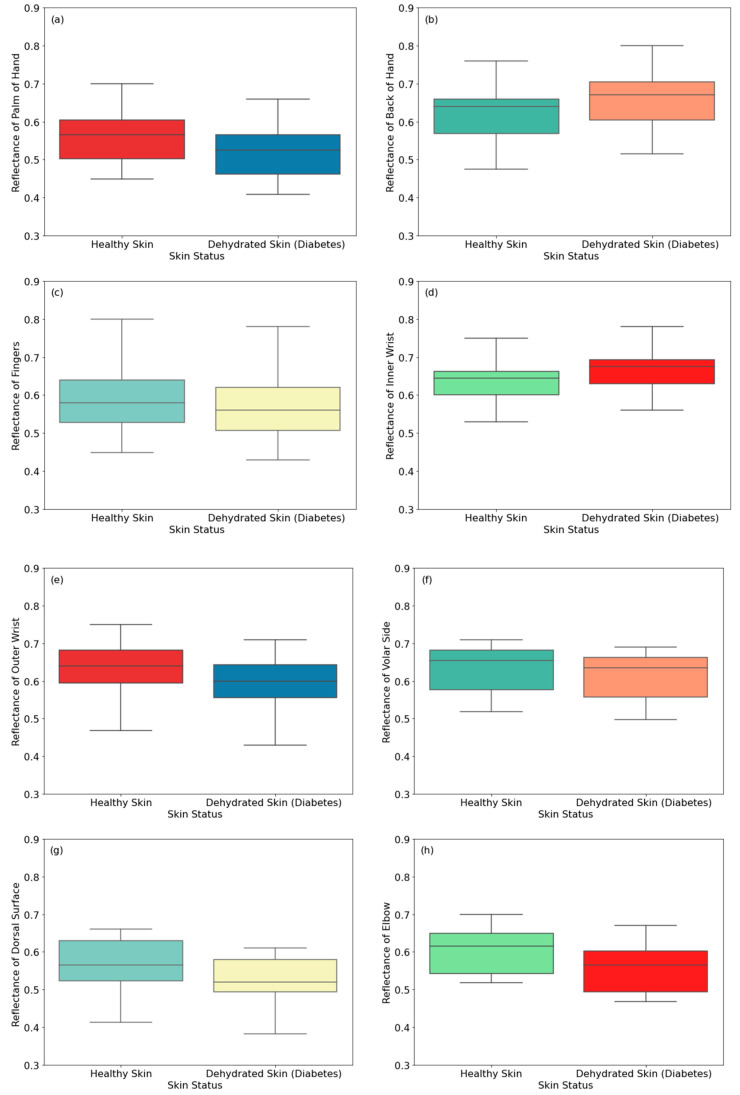
Reflectance measurements of healthy and dehydrated skin (due to diabetes) for female participants on the palm (**a**), the back of the hand (**b**), the fingers (**c**), the inner wrist (**d**), the outer wrist (**e**), the volar side of the arm (**f**), the dorsal surface of the arm (**g**), and the elbow (**h**).

This section presents reflectance measurements of healthy and dehydrated female participants due to diabetes Type 2. The measurements in Figure 9 show reflectance from 20 healthy and 20 dehydrated female participants with Type 2 diabetes. The measurements were applied to eight regions of the skin. Experimental measurements of the reflectance on the back of the hand and inner wrist regions of dehydrated skin are higher than those of healthy skin by 0.041 and 0.030, respectively. These results are consistent with those obtained from male participants in Figure 6. On the other hand, the reflectance of dehydrated skin regions of the palm of the hand, fingers, outer wrist, volar side of the arm, dorsal surface of the arm, and the elbow is lower than that of healthy skin by 0.04, 0.020, 0.04, 0.020, 0.04, and 0.046, respectively.

## 4. Discussion

In this research, reflectance measurements were applied to two groups. Group 1 consisted of 120 male participants (30 healthy, 30 dehydrated due to less thirst sensation, 30 dehydrated due to illnesses, and 30 dehydrated due to diabetes), whereas group 2 consisted of 80 female participants (20 healthy, 20 dehydrated due to less thirst sensation, 20 dehydrated due to illnesses, and 20 dehydrated due to diabetes).

Results obtained from healthy male participants indicated variation in the skin reflectance with measurement locations. Table 2 presents the mean (µ) and the standard deviation (SD) of the reflectance on the eight measured locations on the arm and the hand. The measurements obtained from healthy participants indicate that the reflectance of thinner skin regions, i.e., the back of the hand, the inner wrist, and the volar side, is higher than that of thicker skin regions, i.e., the palm of the hand, the elbow, and the dorsal surface. These results are consistent with the reflectance findings in [33].

Results obtained from dehydrated male participants (due to less thirst sensation and illnesses) indicated lower reflectance measurements for all measurement locations compared with similar regions of healthy skin, as illustrated in Table 2. Elderly patients suffering from vomiting and diarrhea (illnesses) have lower reflectance than those with less thirst sensation and this is due to the illnesses that make the patient lose lots of fluids and suffer from severe dehydration. The differences in the reflectance between dehydrated skin due to less thirst sensation and dehydration due to illnesses are 0.031, 0.033, 0.041, 0.034, 0.025, 0.029, 0.044, and 0.052 for the palm, the back of the hand, the fingers, the inner wrist, the outer wrist, the volar side of the arm, the dorsal surface of the arm, and the elbow, respectively.

Results obtained from dehydrated skin due to diabetes Type 2 indicate that the reflectance for the back of the hand and the inner wrist regions of the skin is higher than that of healthy skin by 0.03 and 0.048, respectively. This is due to diabetes, which affects the thickness of the skin [49,50,51] and makes thinner skin regions more reflective, as a result of blood vessels being closer to the skin surface.

Dehydrated skin reflectance due to vomiting and diarrhea, i.e., illnesses, is lower for all measurement locations compared with healthy and other types of dehydrated skin, i.e., due to less thirst sensation and diabetes. This is due to vomiting and diarrhea, which cause a substantial loss in water content and fluid in the human body. As a result, this affects the human skin reflectance.

Reflectance measurements of the elbow region in this research indicate that this region is very sensitive to variation in reflectance due to dehydration. The elbow shows differences in the reflectance between healthy and dehydrated skin by 16.8%, 22%, and 7% for dehydrated skin due to less thirst sensation, dehydrated skin due to illnesses, and dehydrated skin due to diabetes, respectively.

Reflectance measurements on the female group were consistent with the findings obtained from the male group. The measurements indicate differences in the reflectance between healthy and dehydrated skin, as illustrated in Table 3. In addition, there is variation in reflectance from one location to another as a result of variation in skin thickness [33].

Results obtained from dehydrated female participants (due to less thirst sensation and illnesses) indicated lower reflectance measurements for all measurement locations compared with similar regions of healthy skin, as illustrated in Table 3. The differences in the reflectance between dehydrated skin due to less thirst sensation and dehydration due to illnesses are 0.029, 0.024, 0.028, 0.034, 0.036, 0.020, 0.025, and 0.054 for the palm, the back of the hand, the fingers, the inner wrist, the outer wrist, the volar side of the arm, the dorsal surface of the arm, and the elbow, respectively.

Results obtained from dehydrated skin due to diabetes Type 2 in female participants indicate that the reflectance for the back of the hand and the inner wrist regions of the skin is higher than that of healthy skin by 0.041 and 0.030, respectively. These results are consistent with that obtained from male participants. These results indicate that those two regions might not be suitable to assess skin dehydration for both genders, as they show different trends compared with other measurement locations.

The elbow region showed the highest differences in the reflectance between healthy and dehydrated skin, at 22%, 27.4%, and 4.6% for dehydrated skin due to less thirst sensation, dehydrated skin due to illnesses, and dehydrated skin due to diabetes, respectively. These findings are consistent with those obtained from male participants. This indicates that the elbow region is a suitable region for detecting dehydration of the skin.

## 5. Conclusions

This paper presents reflectance measurements of healthy and dehydrated skin. The reflectance measurements were applied to eight locations on the hand and arm, namely the palm, the back of the hand, the fingers, the inner wrist, the outer wrist, the volar side of the arm, the dorsal surface of the arm, and the elbow. In general, the reflectance of healthy skin is higher than that of dehydrated skin. This is due to water content, which makes healthy skin more reflective compared with dehydrated skin. The measurements indicate substantial differences in the reflectance between healthy skin and dehydrated skin. The paper presents reflectance measurements for dehydrated skin caused by less thirst sensation, illnesses, and diabetes. Elderly patients suffering from vomiting and diarrhea (illnesses) were found to have lower reflectance than those with less thirst sensation. This is due to the illnesses, which make the patient lose lots of fluids and suffer from severe dehydration. For both genders, dehydrated skin due to vomiting and diarrhea was found to have lower reflectance for all measurement locations compared with healthy and other types of dehydrated skin. For male participants, the differences in the reflectance between healthy and dehydrated skin regions (due to illnesses) are 0.110, 0.118, 0.082, 0.075, 0.160, 0.108, 0.156, and 0.220 for the palm of the hand, the back of the hand, the fingers, inner wrist, outer wrist, volar side of the arm, dorsal surface of the arm, and the elbow, respectively, whereas for female participants, the differences are 0.122, 0.076, 0.060, 0.068, 0.195, 0.083, 0.143, and 0.274, respectively. The elbow region shows the highest differences in the reflectance between healthy and dehydrated skin of both genders, and it can be up to 27.4% for female participants and 22% for male participants. These findings indicate that MMW radiometry can be used as a non-contact sensor for early detection of dehydration. As a plan for future work, it is recommended to conduct more measurements on big samples of healthy and dehydrated skin participants, especially from the elderly and children (as they are the categories most affected by dehydration). Then, the mean reflectance values of healthy and dehydrated skin of the same region should be identified. This involves considering the standard deviation of a healthy population, so any deviations from the standard norms should be identified as well as unusually high or low levels of the mean reflectance values. In addition, machine learning classification models should be developed to identify the status of the skin, i.e., healthy or dehydrated, such as K-Nearest Neighbors (KNN), Support Vector Machines (SVM), and Multiple-Layer Perceptron Neural Networks (MLPNNs).

## Figures and Tables

**Table 1:** Participant groups involved in reflectance measurements in this research.

Group Skin Status	Male Number	Age µ ± SD	Female Number	Female µ ± SD
Healthy	30	67.4 ± 7.2	20	65.0 ± 4.4
Dehydrated skin due to less thirst sensation	30	67.6 ± 5.8	20	65.4 ± 6.7
Dehydrated skin due to vomiting	30	67.2 ± 3.3	20	65.9 ± 2.8
Dehydrated skin due to diabetes	30	67.1 ± 6.5	20	65.4 ± 7.6

**Table 2:** The mean and the standard deviation of the reflectance results on healthy and dehydrated skin of male participants.

Location	Healthy µ ± SD	Dehydration (Less Thirst Sensation)	Dehydration (Illnesses)	Dehydration (Diabetes)
Palm of Hand	0.538 ± 0.098	0.458 ± 0.102	0.427 ± 0.104	0.500 ± 0.098
Back of Hand	0.619 ± 0.081	0.534 ± 0.085	0.501 ± 0.084	0.649 ± 0.083
Fingers	0.576 ± 0.114	0.535 ± 0.115	0.494 ± 0.116	0.550 ± 0.114
Inner Wrist	0.613 ± 0.072	0.572 ± 0.072	0.538 ± 0.070	0.661 ± 0.075
Outer Wrist	0.597 ± 0.063	0.465 ± 0.071	0.440 ± 0.070	0.538 ± 0.065
Volar Side	0.624 ± 0.075	0.544 ± 0.073	0.515 ± 0.075	0.572 ± 0.078
Dorsal Surface	0.559 ± 0.082	0.447 ± 0.083	0.403 ± 0.0807	0.524 ± 0.083
Elbow	0.588 ± 0.075	0.420 ± 0.083	0.368 ± 0.087	0.518 ± 0.072

**Table 3:** The mean and the standard deviation of the reflectance results on healthy and dehydrated skin of female participants.

Location	Healthy µ ± SD	Dehydration (Less Thirst Sensation)	Dehydration (Illnesses)	Dehydration (Diabetes)
Palm of Hand	0.566 ± 0.074	0.473 ± 0.068	0.444 ± 0.069	0.5262 ± 0.075
Back of Hand	0.625 ± 0.076	0.573 ± 0.066	0.549 ± 0.064	0.666 ± 0.076
Fingers	0.584 ± 0.091	0.552 ± 0.092	0.524 ± 0.083	0.564 ± 0.091
Inner Wrist	0.634 ± 0.057	0.600 ± 0.054	0.566 ± 0.0532	0.664 ± 0.057
Outer Wrist	0.633 ± 0.066	0.474 ± 0.064	0.438 ± 0.065	0.593 ± 0.066
Volar Side	0.633 ± 0.061	0.570 ± 0.0586	0.550 ± 0.061	0.613 ± 0.061
Dorsal Surface	0.563 ± 0.068	0.446 ± 0.095	0.421 ± 0.094	0.523 ± 0.0644
Elbow	0.603 ± 0.060	0.383 ± 0.067	0.329 ± 0.067	0.557 ± 0.062

## Data Availability

Data are contained within the article.

## References

[1] Aging UK Later Life in the United Kingdom. https://www.ageuk.org.uk/globalassets/age-uk/documents/reports-and-publications/later_life_uk_factsheet.pdf.

[2] Kubala J., Sissons C. What is the Average Percentage of Water in the Human Body?. https://www.medicalnewstoday.com/articles/what-percentage-of-the-human-body-is-water#why-water-is-important.

[3] Stookey D.J. (2010). Drinking Water and Weight Management. Nutr. Today.

[4] Pan A., Malik V.S., Hao T., Willett W.C., Mozaffarian D., Hu F.B. (2013). Changes in water and beverage intake and long-term weight changes: Results from three prospective cohort studies. Int. J. Obes..

[5] García A.I.L., Moráis-Moreno C., Samaniego-Vaesken M.d.L., Puga A.M., Varela-Moreiras G., Partearroyo T. (2019). Association between Hydration Status and Body Composition in Healthy Adolescents from Spain. Nutrients.

[6] Muckelbauer R., Sarganas G., Grüneis A., Müller-Nordhorn J. (2013). Association between water consumption and body weight outcomes: A systematic review. Am. J. Clin. Nutr..

[7] Bar-David Y., Urkin J., Kozminsky E. (2005). The effect of voluntary dehydration on cognitive functions of elementary school children. Acta Paediatr..

[8] Popkin B.M., D’Anci K.E., Rosenberg I.H. (2010). Water, hydration, and health. Nutr. Rev..

[9] Shaheen N.A., Alqahtani A.A., Assiri H., Alkhodair R., Hussein M.A. (2018). Public knowledge of dehydration and fluid intake practices: Variation by participants’ characteristics. BMC Public Health.

[10] Nagae M., Umegaki H., Komiya H., Fujisawa C., Watanabe K., Yamada Y., Miyahara S. (2022). Dehydration and hospital-associated disability in acute hospitalized older adults. Eur. Geriatr. Med..

[11] Atciyurt K., Heybeli C., Smith L., Veronese N., Soysal P. (2024). The prevalence, risk factors and clinical implications of dehydration in older patients: A cross-sectional study. Acta Clin. Belg..

[12] Falszewska A., Szajewska H., Dziechciarz P. (2018). Diagnostic accuracy of three clinical dehydration scales: A systematic review. Arch. Dis. Child..

[13] Collins M., Claros E. (2011). Recognizing the face of dehydration. Nursing2021.

[14] Watso J.C., Farquhar W.B. (2019). Hydration Status and Cardiovascular Function. Nutrients.

[15] Hurlow J., Bliss D.Z. (2011). Dry Skin in Older Adults. Geriatr. Nurs..

[16] Honari G., Maibach H. (2014). Skin Structure and Function. Applied Dermatotoxicology.

[17] Owda A.Y., Salmon N., Harmer S.W., Shylo S., Bowring N.J., Rezgui N.D., Shah M. (2017). Millimeter-wave emissivity as a metric for the non-contact diagnosis of human skin conditions. Bioelectromagnetics.

[18] Saranya K., Vijayashaarathi S., Sasirekha N., Rishika M., Rajeswari P.S.R. Skin Disease Detection Using CNN (Convolutional Neural Network). Proceedings of the 2024 4th International Conference on Data Engineering and Communication Systems (ICDECS).

[19] Eda N., Nakamura N., Inai Y., Sun Z., Sone R., Watanabe K., Akama T. (2022). Changes in the skin characteristics associated with dehydration and rehydration. Eur. J. Sport Sci..

[20] Rodrigues L.M., Palma L., Marques L.T., Varela J.B. (2015). Dietary water affects human skin hydration and biomechanics. Clin. Cosmet. Investig. Dermatol..

[21] Hooper L., Bunn D.K., Abdelhamid A., Gillings R., Jennings A., Maas K., Millar S., Twomlow E., Hunter P.R., Shepstone L. (2016). Water-loss (intracellular) dehydration assessed using urinary tests: How well do they work? Diagnostic accuracy in older people. Am. J. Clin. Nutr..

[22] Hew-Butler T.D., Eskin C., Bickham J., Rusnak M., VanderMeulen M. (2018). Dehydration is how you define it: Comparison of 318 blood and urine athlete spot checks. BMJ Open Sport Exerc. Med..

[23] Crosignani A., Spina S., Marrazzo F., Cimbanassi S., Malbrain M.L.N.G., Van Regenemortel N., Fumagalli R., Langer T. (2022). Intravenous fluid therapy in patients with severe acute pancreatitis admitted to the intensive care unit: A narrative review. Ann. Intensiv. Care.

[24] Gawronska J., Koyanagi A., Sánchez G.F.L., Veronese N., Ilie P.C., Carrie A., Smith L., Soysal P. (2022). The Prevalence and Indications of Intravenous Rehydration Therapy in Hospital Settings: A Systematic Review. Epidemiologia.

[25] Gidado I.M., Qassem M., Triantis I.F., Kyriacou P.A. (2022). Review of Advances in the Measurement of Skin Hydration Based on Sensing of Optical and Electrical Tissue Properties. Sensors.

[26] Ruini C., Kendziora B., Ergun E.Z., Sattler E., Gust C., French L.E., Bağcı I.S., Hartmann D. (2021). In vivo examination of healthy human skin after short-time treatment with moisturizers using confocal Raman spectroscopy and optical coherence tomography: Preliminary observations. Ski. Res. Technol..

[27] Qassem M., Kyriacou P. (2019). Review of Modern Techniques for the Assessment of Skin Hydration. Cosmetics.

[28] Mamouei M., Chatterjee S., Razban M., Qassem M., Kyriacou P.A. (2021). Design and Analysis of a Continuous and Non-Invasive Multi-Wavelength Optical Sensor for Measurement of Dermal Water Content. Sensors.

[29] Flament F., Galliano A., Abric A., Matoschitz C., Bammer M., Kampus M., Kanda-Diwidi D., Chibout S., Cassier M., Delaunay C. (2021). Skin moisture assessment using Hydration Sensor Patches coupled with smartphones via Near Field Communication (NFC). A pilot study with the first generation of patches that allow self-recordings of skin hydration. Ski. Res. Technol..

[30] Matsukawa R., Miyamoto A., Yokota T., Someya T. (2020). Skin Impedance Measurements with Nanomesh Electrodes for Monitoring Skin Hydration. Adv. Health Mater..

[31] Westermann T.V.A., Viana V.R., Junior C.B., da Silva C.B.D., Carvalho E.L.S., Pupe C.G. (2020). Measurement of skin hydration with a portable device (SkinUp^®^ Beauty Device) and comparison with the Corneometer^®^. Ski. Res. Technol..

[32] Smulders P. (2012). Analysis of human skin tissue by millimeter-wave reflectometry. Ski. Res. Technol..

[33] Owda A.Y., Salmon N., Casson A.J., Owda M. (2020). The Reflectance of Human Skin in the Millimeter-Wave Band. Sensors.

[34] Gao Y., Zoughi R. (2016). Millimeter Wave Reflectometry and Imaging for Noninvasive Diagnosis of Skin Burn Injuries. IEEE Trans. Instrum. Meas..

[35] Alekseev S., Ziskin M. (2007). Human skin permittivity determined by millimeter wave reflection measurements. Bioelectromagnetics.

[36] Alabaster C. (2003). Permittivity of human skin in millimetre wave band. Electron. Lett..

[37] Zhadobov M., Chahat N., Sauleau R., Le Quement C., Le Drean Y. (2011). Millimeter-wave interactions with the human body: State of knowledge and recent advances. Int. J. Microw. Wirel. Technol..

[38] Lubecke O.B., Nikawa Y., Snyder W., Lin J., Mizuno K. Novel microwave and millimeter-wave biomedical applications. Proceedings of the 4th International Conference, In Telecommunications in Modern Satellite, Cable and Broadcasting Services, TELSIKS’99 (Cat. No.99EX365).

[39] Wallace V., Fitzgerald A., Shankar S., Flanagan N., Pye R., Cluff J., Arnone D. (2004). Terahertz pulsed imaging of basal cell carcinoma ex vivo and in vivo. Br. J. Dermatol..

[40] Owda A.Y., Owda M. (2024). Homogenous and multilayer electromagnetics models for estimating skin reflectance. Indones. J. Electr. Eng. Comput. Sci..

[41] Harmer S.W., Shylo S., Shah M., Bowring N.J., Owda A.Y. (2016). On the feasibility of assessing burn wound healing without removal of dressings using radiometric millimetre-wave sensing. Prog. Electromagn. Res. M.

[42] Owda A.Y. (2022). Passive Millimeter-Wave Imaging for Burns Diagnostics under Dressing Materials. Sensors.

[43] Salmon N.A. (2018). Outdoor Passive Millimeter-Wave Imaging: Phenomenology and Scene Simulation. IEEE Trans. Antennas Propag..

[44] Páez A., Boisjoly G. (2022). Exploratory Data Analysis. Discrete Choice Analysis with R.

[45] Appleby R., Anderton R.N. (2007). Millimeter-Wave and Submillimeter-Wave Imaging for Security and Surveillance. Proc. IEEE.

[46] Załęcki P., Rogowska K., Wąs P., Łuczak K., Wysocka M., Nowicka D. (2024). Impact of Lifestyle on Differences in Skin Hydration of Selected Body Areas in Young Women. Cosmetics.

[47] Bossingham M.J., Carnell N.S., Campbell W.W. (2005). Water balance, hydration status, and fat-free mass hydration in younger and older adults. Am. J. Clin. Nutr..

[48] Li S., Xiao X., Zhang X. (2023). Hydration Status in Older Adults: Current Knowledge and Future Challenges. Nutrients.

[49] Derraik J.G.B., Rademaker M., Cutfield W.S., Pinto T.E., Tregurtha S., Faherty A., Peart J.M., Drury P.L., Hofman P.L. (2014). Effects of Age, Gender, BMI, and Anatomical Site on Skin Thickness in Children and Adults with Diabetes. PLoS ONE.

[50] Collier A., Patrick A.W., Bell D., Matthews D.M., Maclntyre C.C.A., Ewing D.J., Clarke B.F. (1989). Relationship of Skin Thickness to Duration of Diabetes, Glycemic Control, and Diabetic Complications in Male IDDM Patients. Diabetes Care.

[51] Ruiz-Alejos A., Carrillo-Larco R.M., Miranda J.J., Gilman R.H., Smeeth L., Bernabé-Ortiz A. (2019). Skinfold thickness and the incidence of type 2 diabetes mellitus and hypertension: An analysis of the PERU MIGRANT study. Public Health Nutr..

